# Engineering Bacteria as Living Therapeutics in Cancer Therapy

**DOI:** 10.1002/advs.202507820

**Published:** 2025-08-28

**Authors:** Jiangfeng Chen, Jiaqi Chen, Yixuan Chen, Wenxuan Yuan, Jiao Zhang, Guoqing Wang, Zhuojun Dai

**Affiliations:** ^1^ College of Basic Medical Science Jilin University Changchun 130021 P. R. China; ^2^ Key Laboratory of Quantitative Synthetic Biology Shenzhen Institute of Synthetic Biology Shenzhen Institutes of Advanced Technology Chinese Academy of Sciences Shenzhen 518000 P. R. China; ^3^ Department of Biomedical Sciences Faculty of Health Sciences University of Macau Taipa Macau SAR 999078 P. R. China; ^4^ University of Chinese Academy of Sciences Beijing 100049 P. R. China

**Keywords:** cancer immunotherapy, engineered bacteria, synthetic biology, targeted drug delivery, tumor microenvironment modulation

## Abstract

Cancer is the second‐leading cause of death globally, yet traditional therapies like chemotherapy face significant limitations. Recent advances in synthetic biology enable the design of various genetic circuits and the reprogramming of biological systems. Collectively, these efforts have led to the repurposing of engineered bacteria as therapeutics to achieve tumor targeting, tumor microenvironment modulation, and anticancer drug release. Here, these recent efforts are reviewed and discussed the challenges and future opportunities.

## Graphical abstract

Through synthetic biology approaches, natural bacteria can be genetically programmed into multifunctional living therapeutics. These engineered bacteria are capable of secreting anti‐tumor factors, displaying tumor‐associated antigens on their surface, or undergoing programmed self‐lysis to release therapeutic cargo. These functions are precisely orchestrated by the genetic circuits, enabling spatiotemporal control of therapeutic response.

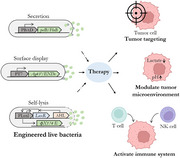

## 1 Introduction

Cancer remains one of the most common causes of death worldwide, claiming the lives of approximately nine million individuals annually.^[^ ^1^ ^]^ A critical focus of modern scientific research lies in discovering novel therapies that selectively target and eliminate cancer cells while sparing healthy tissues. However, achieving this precision remains a significant hurdle in medical and biological science.^[^ ^2^ ^]^ For example, chemotherapy is a frequently used method in cancer control but is associated with significant systemic toxicity and severe adverse effects due to the drug's non‐selective action on both cancerous and healthy cells. These drugs can induce long‐term or irreversible organ damage. Resistance often emerges and reduces the effectiveness of chemotherapy, leading to treatment failure, disease progression, and diminished long‐term survival outcomes.^[^ ^3^, ^4^ ^]^ Therefore, there is a pressing need to discover a cancer therapy with high precision, reduced side effects, and resistance resilience.

Research into bacterial cancer therapy can be traced back to the late 19th century. William B. Coley, a New York surgeon, noticed an interesting case from the hospital's records: a patient who initially after contracting erysipelas had a tumor regression.^[^ ^5^ ^]^ This observation inspired him to hypothesize that infections might induce tumor remission. In 1891, William B. Coley intentionally injected his patient with *Streptococcus pyogenes* and *Serratia marcescens* to treat cancer and observed a substantial tumor regression.^[^ ^6^ ^]^ This work pioneered immunotherapy before the modern understanding of the immune system. The drug (Coley's toxin) was commercially produced in 1899 and used for 30 years for in treating inoperable bone and soft‐tissue sarcomas, before Food and Drug Administration questioned its working mechanism and safety.^[^ ^7^ ^]^

We now know that bacteria can stimulate the immune system.^[^ ^8^ ^]^ They also preferentially grow in hypoxic and immunosuppressive tumor microenvironments. This unique adaptability allows them to both amplify antitumor immune responses and colonize hostile tumor regions.^[^ ^9^ ^]^ Furthermore, bacterial growth produces metabolites that can directly influence tumor cells and reshape the surrounding microenvironment.^[^ ^10^, ^11^ ^]^ Collectively, this dynamic interplay positions bacteria as a promising therapeutic modality for cancer treatment.

Over the last two decades, synthetic biology has made significant strides, enabling scientists to program living cells for system investigation and manipulate cells to productive ends.^[^ ^12^ ^]^ As an engineering discipline, synthetic biology expands the subject matter of biology from the space of existing species and cellular systems to the even larger space of non‐natural, but feasible species and systems.^[^ ^13^ ^]^ To date, the area has accumulated a library of well‐characterized parts, regulatory genetic circuits, and system design approaches, serving as the foundation for programming cells to accomplish diverse tasks.^[^ ^14^, ^15^ ^]^ These advancements have brought the potential to develop safer and more effective engineered bacteria therapies, by enabling control over the delivery and dosing of therapeutic activities in a dynamic temporal and spatial manner (**Table** **1**).

**Table 1:** Representative studies of engineered bacteria for cancer therapy.

Number	Year	Article title	Mechanism of tumor therapy	DOI
1	2000	Bifidobacterium longum as a delivery system for cancer gene therapy: selective localization and growth in hypoxic tumors.^[^ ^16^ ^]^	Immune modulation.	10.1038/sj.cgt.7700122
2	2006	Targeted therapy with a *Salmonella typhimurium* leucine‐arginine auxotroph cures orthotopic human breast tumors in nude mice.^[^ ^17^ ^]^	Expression of oncolysins and immune activation factors.	10.1158/0008‐5472.CAN‐06‐0716
3	2016	Synchronized cycles of bacterial lysis for in vivo delivery.^[^ ^11^ ^]^	Release of oncolytic factors and immune activation factors.	10.1038/nature18930
4	2018	Tumor targeting *Salmonella typhimurium* A1‐R in combination with gemcitabine (GEM) regresses partially GEM‐resistant pancreatic cancer patient‐derived orthotopic xenograft (PDOX) nude mouse models.^[^ ^18^ ^]^	Oncolytic effect and immune activation.	10.1080/15384101.2018.1480223
5	2019	Programmable bacteria induce durable tumor regression and systemic antitumor immunity.^[^ ^19^ ^]^	Oncolytic effect and immune activation.	10.1038/s41591‐019‐0498‐z
6	2020	Engineered probiotics for local tumor delivery of checkpoint blockade nanobodies.^[^ ^20^ ^]^	Local treatment and immune activation.	10.1126/scitranslmed.aax0876
7	2020	Immunotherapy with engineered bacteria by targeting the STING pathway for anti‐tumor immunity.^[^ ^21^ ^]^	Activate the immune microenvironment.	10.1038/s41467‐020‐16602‐0
8	2022	A programmable encapsulation system improves the delivery of therapeutic bacteria in mice.^[^ ^22^ ^]^	Expression of oncolysins, activation of the immune system.	10.1038/s41587‐022‐01244‐y
9	2023	Chemokines expressed by engineered bacteria recruit and orchestrate antitumor immunity.^[^ ^23^ ^]^	Activating immune responses.	10.1126/sciadv.adc9436
10	2023	Engineered skin bacteria induce antitumor T cell responses against melanoma.^[^ ^24^ ^]^	Activating immune responses.	10.1126/science.abp9563
11	2024	Engineering tumor‐colonizing *E. coli* Nissle 1917 for detection and treatment of colorectal neoplasia.^[^ ^25^ ^]^	Expressing tumor‐killing substances.	10.1038/s41467‐024‐44776‐4
12	2024	Prodrug‐conjugated tumor‐seeking commensals for targeted cancer therapy.^[^ ^26^ ^]^	Expressing tumor‐killing substances.	10.1038/s41467‐024‐48661‐y
13	2024	Sonogenetics‐controlled synthetic designer cells for cancer therapy in tumor mouse models.^[^ ^27^ ^]^	Reprogramming the immune system.	10.1016/j.xcrm.2024.101513
14	2024	Reprogramming the tumor immune microenvironment using engineered dual‐drug loaded *Salmonella*.^[^ ^28^ ^]^	Regulating the tumor microenvironment.	10.1038/s41467‐024‐50950‐5
15	2024	High‐Lactate‐Metabolizing photosynthetic bacteria reprogram TIM.^[^ ^29^ ^]^	Regulating the tumor microenvironment.	10.1002/adma.202405930
16	2024	Programmable bacteria synergize with PD‐1 blockade to overcome cancer cell‐intrinsic immune resistance mechanisms.^[^ ^30^ ^]^	Activating immune responses.	10.1126/sciimmunol.adn9879
17	2024	Non‐pathogenic *E. coli* displaying decoy‐resistant IL18 mutein boosts anti‐tumor and CAR NK cell responses.^[^ ^31^ ^]^	Activating immune responses.	10.1038/s41587‐024‐02418‐6
18	2024	Probiotic neoantigen delivery vectors for precision cancer immunotherapy.^[^ ^32^ ^]^	Activating immune responses.	10.1038/s41586‐024‐08033‐4
19	2024	Hybridized and engineered microbe for catalytic generation of peroxynitrite and cancer immunotherapy under sonopiezo initiation.^[^ ^33^ ^]^	Expressing tumor‐killing substances.	10.1126/sciadv.adp7540
20	2024	Combinatorial leaky probiotic for anticancer immunopotentiation and tumor eradication.^[^ ^34^ ^]^	Expressing tumor‐killing substances.	10.1016/j.xcrm.2024.101793
21	2025	Bacterial immunotherapy leveraging IL‐10R hysteresis for both phagocytosis evasion and tumor immunity revitalization.^[^ ^35^ ^]^	Activating immune responses.	10.1016/j.cell.2025.02.002
22	2025	Engineered bacteria manipulate cysteine metabolism to boost ferroptosis‐based pancreatic ductal adenocarcinoma therapy.^[^ ^36^ ^]^	Expressing tumor‐killing substances.	10.1002/adma.202412982
23	2025	Engineered bacteria for near‐infrared light‐inducible expression of cancer therapeutics.^[^ ^37^ ^]^	Expressing tumor‐killing substances.	10.1038/s43018‐025‐00932‐3
24	2025	Programmable engineered bacteria as sustained‐releasing antibody factory in situ for enhancing tumor immune checkpoint therapy.^[^ ^38^ ^]^	Bacterial colonization with immune system activation.	10.1126/sciadv.adt7298
25	2025	An antibody‐toxin conjugate targeting CD47 linked to the bacterial toxin listeriolysin O for cancer immunotherapy.^[^ ^39^ ^]^	Expressing tumor‐killing substances.	10.1038/s43018‐025‐00919‐0
26	2025	Aggregation induced emission luminogen bacteria hybrid bionic robot for multimodal phototheranostics and immunotherapy.^[^ ^40^ ^]^	Bacterial colonization with immune system activation.	10.1038/s41467‐025‐57533‐y
27	2025	In situ production and precise release of bioactive GM‐CSF and siRNA by engineered bacteria for macrophage reprogramming in cancer immunotherapy.^[^ ^41^ ^]^	Bacterial colonization with immune system activation.	10.1016/j.biomaterials.2024.123037
28	2025	Targeted tumor therapy with L‐cyst(e)ine‐addicted bacteria‐nanodrug biohybrids.^[^ ^42^ ^]^	Expressing tumor‐killing substances.	10.1016/j.cmet.2025.03.012

In this review, we outline recent advances in the use of engineered bacteria for cancer therapy. While several prior reviews have systematically summarized the development of bacterial derivatives—such as outer membrane vesicles, biohybrid platforms, nanoparticles, and delivery strategies^[^ ^43^, ^44^, ^45^, ^46^, ^47^ ^]^—these works primarily focus on non‐living systems and material‐based carriers. In contrast, our review emphasizes living bacterial therapeutics, with a particular focus on dynamic, stimulus‐responsive design principles and integrative immune engineering strategies. By highlighting these emerging approaches, we aim to complement existing literature and provide new insights into the design of programmable, tumor‐adaptive bacterial therapeutics.

## 2 Engineered Bacteria Therapy

### 2.1 Tumor Targeting

#### 2.1.1 Naturally Occurred Cell Strain

**Figure 1:**
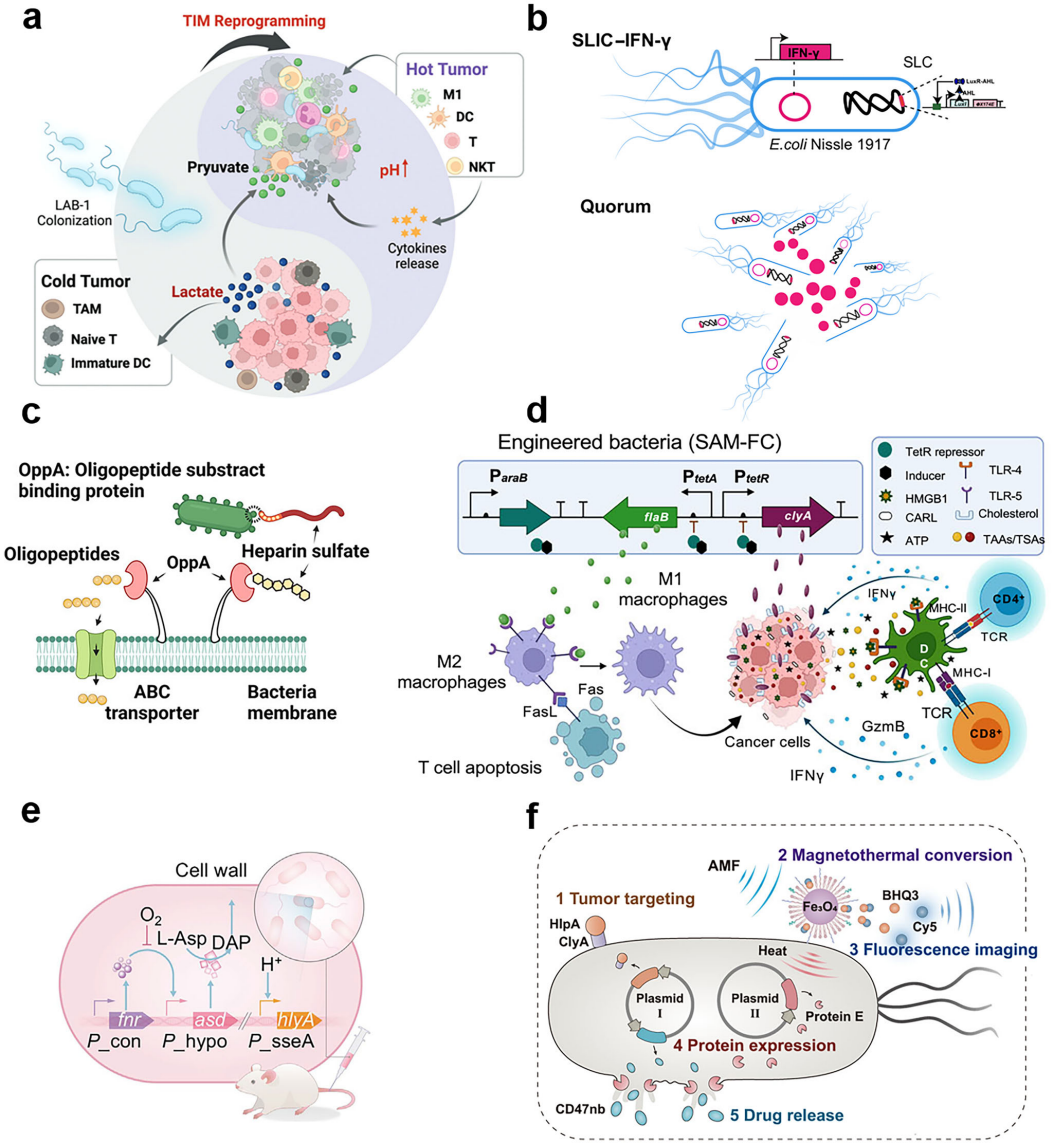
Tumor‐targeting capabilities of living bacteria. a) Colonization of natural bacteria strain. *R. palustris* LAB‐1 selectively colonizes the hypoxic TME, where it depletes lactate by converting it into pyruvate via native lactate dehydrogenase, supporting facultative anaerobic metabolism. This process not only enables selective colonization in lactate‐rich, hypoxic regions but also mitigates immunosuppression, promoting a more immuno‐active TME. The image is reproduced from ref.[29] The image is reproduced with permission from Ma *et al.*, *Advanced Science*, 2024, 36, adc2405930. 2024 Wiley‐VCH. [Reprinted under license number 6016991182106]. b) Colonization of natural probiotics. Intravenously administered *E. coli* Nissle 1917 was engineered with a chromosomally integrated quorum‐sensing circuit and a SLC, enabling population‐wide lysis upon reaching critical density. This strategy facilitated the localized release of genetically encoded IFN‐γ and PAMPs, thereby promoting immune activation and suppressing tumor growth. The image is reproduced from ref.[30]. The image is reproduced with permission from Li *et al.*, *Science immunology*, 2024, 9, adc9879. 2024 American Association for the Advancement of Science. [Reprinted under license number 6016990521644]. c) Naturally occurred tumor‐targeting proteins on bacteria surface. A strain of *L. plantarum* WCFS1, displaying the oligopeptide‐binding protein OppA on its surface, specifically targets nasopharyngeal carcinoma cells via high‐affinity interaction with overexpressed heparan sulfate glycosaminoglycans on the tumor membrane. The image is reproduced from ref.[26] The image is adapted from Shen *et al.*, *Nature Communications*, 2024, 15, adc4343. 2024 The Author(s). licensed under a Creative Commons Attribution 4.0 International License (CC BY 4.0). https://doi.org/10.1038/s41467‐024‐48661‐y. d) Genetic attenuation of bacterial virulence. Intratumoral injection of *S. typhimurium*, attenuated by deletion of 12 GGDEF domain genes, was combined with a tetracycline‐inducible system to express the immunomodulatory proteins ClyA and FlaB. This approach enhanced antigen presentation, reprogrammed M2 macrophages toward a pro‐inflammatory M1 phenotype, and reshaped the tumor microenvironment, resulting in effective clearance of primary and metastatic tumors. The image is reproduced from ref.[28] The image is adapted from Nguyen *et al.*, *Nature Communications*, 2024, 15, adc6680. 2024 The Author(s). licensed under a Creative Commons Attribution 4.0 International License (CC BY 4.0). https://doi.org/ 10.1038/s41467‐024‐50950‐5. e) Tumor colonization by engineered hypoxic bacteria. The *S. typhimurium* strain DB1 was engineered by placing the essential *asd* gene under a hypoxia‐inducible promoter (P_hypo_) for selective survival in hypoxic tumor regions. An inducible *hlyA* gene enhanced bacterial translocation and colonization. DB1's immunogenicity activated TAMs, induced IL‐10 secretion, and reshaped the tumor immune microenvironment. The image is reproduced from ref.[35] The image is reproduced with permission from Chang *et al.*, *Cell*, 2025, 188, adc1‐16. 2025 Elsevier Inc. [Reprinted under license number 6017000712717]. f) Engineering probiotics with membrane‐tethered targeting proteins. *E. coli* MG1655 was engineered to display the tumor‐targeting protein HlpA on its outer membrane via ClyA fusion, enabling specific binding to HSPG on tumor cells. This facilitated targeted delivery of HlpA and CD47 nanobody, restoring macrophage phagocytosis and enhancing antitumor immunity. The image is reproduced from ref.[61] The image is adapted from Nguyen *et al.*, *Nature Communications*, 2023, 14, adc1606. 2023 The Author(s). Licensed under CC BY 4.0. https://doi.org/10.1038/s41467‐023‐37225‐1.

Tumor microenvironment (TME) is marked by hypoxia and immunosuppression due to rapid tumor proliferation and abnormal vasculature that together restrict oxygen supply.^[^ ^48^, ^49^ ^]^ Meanwhile, tumor cells secrete immunosuppressive factors, express immune checkpoint molecules, and recruit suppressive immune cells, collectively forming a protective niche for immune evasion.^[^ ^50^ ^]^ Certain bacteria can selectively accumulate in tumors due to their chemotactic responses to tumor‐derived signals and their capacity to survive in hostile conditions such as hypoxia and low pH, reaching concentrations up to 10000‐fold higher than in normal tissues.^[^ ^9^, ^19^, ^51^ ^]^ For instance, Ma et al. identified LAB‐1, a photosynthetic *Rhodopseudomonas palustris* strain that thrives in hypoxic, lactate‐rich environments by utilizing lactate as its sole carbon source. Upon intratumoral injection, LAB‐1 selectively colonized tumor tissues and depleted lactate, thereby alleviating immunosuppression and inhibiting tumor growth (Figure **1**a).^[^ ^29^ ^]^ The finding supports that certain bacteria can exploit tumor‐specific biochemical and environmental cues for selective colonization, thereby enhancing therapeutic precision while minimizing toxicity to normal tissues.

Bacterial tumor‐targeting colonization can also be achieved through molecular recognition between naturally expressed bacterial surface proteins and tumor‐specific membrane molecules, thereby reducing off‐target effects on normal tissues.^[^ ^9^ ^]^ For example, Shen et al. identified a therapeutically promising oral commensal strain, *Lactobacillus plantarum* WCFS1 (Lp), for targeting nasopharyngeal carcinoma (NPC).^[^ ^26^ ^]^ Through experimental validation, the authors found that the strain's naturally expressed oligopeptide‐binding protein oligopeptide‐binding protein A (OPPA) selectively bound to heparan sulfate, a glycosaminoglycan highly expressed on NPC cell membranes, thereby conferring tumor‐specific colonization capability to Lp (Figure 1c). Based on this mechanism, the researchers further anchored a biotinylated prodrug, TL‐SN, onto a tetrameric streptavidin complex displayed on the bacterial surface. Upon tumor colonization, reactive oxygen species (ROS) within the tumor microenvironment triggered prodrug activation, releasing the cytotoxic compound SN‐38, and achieving targeted antitumor effects with a tumor inhibition rate of up to 67%. This study demonstrates that leveraging the specific interaction between bacterial surface proteins and tumor‐associated glycan structures enables both selective colonization and localized drug release, thereby enhancing therapeutic precision and minimizing systemic toxicity.

The use of naturally non‐pathogenic probiotic strains capable of surviving and proliferating within tumor‐specific microenvironments—characterized by low pH, hypoxia, and immunosuppression—offers another efficient and targeted strategy for tumor colonization.^[^ ^9^, ^19^ ^]^ When delivered via intratumoral injection, such bacteria can achieve localized enrichment while minimizing off‐target distribution and toxicity.^[^ ^20^ ^]^ Li et al. employed this approach by administering the probiotic strain *Escherichia coli* Nissle 1917 (EcN) into MC38 and CT26 colorectal cancer mouse models.^[^ ^30^ ^]^ Leveraging EcN's inherent ability to colonize immune‐excluded tumor environments, the authors achieved precise bacterial accumulation within tumor tissues. Building on this, a synchronized lysis circuit (SLIC) based on quorum sensing was engineered into the strain. Upon reaching a critical bacterial density, the circuit was activated to induce coordinated lysis, releasing interferon‐gamma (IFN‐γ) along with pathogen‐associated molecular patterns (PAMPs) (Figure 1b), thereby enhancing local antitumor immunity and suppressing tumor growth. This strategy significantly improved therapeutic efficacy while avoiding the systemic inflammation and tissue damage often associated with IFN‐γ administration, highlighting the safety and precision of probiotic‐based tumor immunotherapy.

#### 2.1.2 Engineered Cell Strain

However, certain bacterial strains can still survive in normal tissues, necessitating genetic attenuation to reduce their virulence and endotoxin‐related toxicity.^[^ ^52^ ^]^ For example, Nguyen et al. addressed this by deleting 12 genes encoding GGDEF domain‐containing proteins responsible for the synthesis of the bacterial second messenger cyclic‐di‐GMP in *S. typhimurium*.^[^ ^28^ ^]^ As c‐di‐GMP plays a key role in regulating biofilm formation, chemotaxis, and pathogenicity, its removal significantly reduced the bacterium's virulence while preserving its tumor‐targeting and therapeutic capabilities,^[^ ^53^, ^54^ ^]^ resulting in the construction of an attenuated *S. typhimurium* strain termed SAM‐FC. Furthermore, a tetracycline‐inducible expression system was engineered into the strain to control the expression of cytolysin A (ClyA) and flagellin B (FlaB), two immune‐modulatory proteins (Figure 1d). Upon induction, SAM‐FC was able to modulate the tumor immune microenvironment and achieved 75–80% complete tumor regression in CT26 and MC38 murine tumor models.

Insertion of hypoxia‐responsive gene circuits into bacteria, such as low‐oxygen‐inducible promoters like P_pepT_ or P_hypo_, represents another effective gene regulation strategy to enhance bacterial targeting and colonization in hypoxic tumor regions while restricting their survival in oxygen‐rich normal tissues.^[^ ^20^, ^55^, ^56^ ^]^ Chang et al. engineered an attenuated *S. typhimurium* strain, DB1, by placing the *asd* gene encoding aspartate‐semialdehyde dehydrogenase under the control of the P_hypo_ promoter, thereby adapting bacterial survival and proliferation to hypoxic tumor environments and ensuring rapid clearance from normoxic tissues.^[^ ^35^ ^]^ In addition, the *hlyA* gene, encoding Listeriolysin O, was placed under the control of an intracellularly inducible promoter to enhance bacterial translocation across host cell membranes (Figure 1e). Upon tumor colonization, the immunogenicity of DB1 activated Toll‐like receptor 4 (TLR4) signaling in tumor‐associated macrophages (TAMs), promoting interleukin‐10 (IL‐10) secretion and reshaping the tumor immune microenvironment. This strategy significantly suppressed tumor growth in mouse models of MB49 bladder cancer, B16‐F10 melanoma, and colitis‐associated colorectal cancer.

During tumor progression, genetic mutations, aberrant signaling, and microenvironmental adaptations often lead to the overexpression or abnormal presentation of specific surface proteins, such as receptors, glycosylated antigens, and adhesion molecules, which support tumor proliferation and invasion, immune evasion, and metabolic reprogramming.^[^ ^57^, ^58^, ^59^ ^]^ Leveraging these differences between tumor and normal cells, synthetic biologists engineer the bacterial strain to display tumor‐targeting ligands for precise colonization within tumor tissues.^[^ ^19^, ^60^ ^]^ In a study by Ma et al., *E. coli* MG1655 was engineered to express histone‐like protein A (HlpA) on its outer membrane, which specifically binds to heparan sulfate proteoglycans (HSPG) abundantly expressed on tumor cells.^[^ ^61^ ^]^ HlpA was fused with membrane‐anchoring proteins ClyA or ice nucleation protein and expressed via an exogenous plasmid. Based on this targeting mechanism, the engineered *E. coli* MG1655 strain was further equipped with anti‐CD47 nanobodies (CD47nb) to locally modulate the tumor immune microenvironment (Figure 1f). This strategy achieved a tumor inhibition rate of up to 94.7% in the CT‐26‐luc murine colorectal cancer model.

Engineered strains enhance safety and precision through virulence attenuation (e.g., c‐di‐GMP deletion in *S. typhimurium* SAM‐FC), hypoxia‐responsive circuits (e.g., promoter P_hypo_‐controlled *asd* gene in *S. typhimurium* DB1), and synthetic tumor‐targeting ligands (e.g., HlpA‐HSPG binding in *E. coli* MG1655). These strategies enable localized therapeutic payload delivery (e.g., IFN‐γ, nanobodies) while minimizing systemic toxicity, achieving desirable tumor inhibition rates in preclinical models.

### 2.2 Tumor Microenvironment Modulation

**Figure 2:**
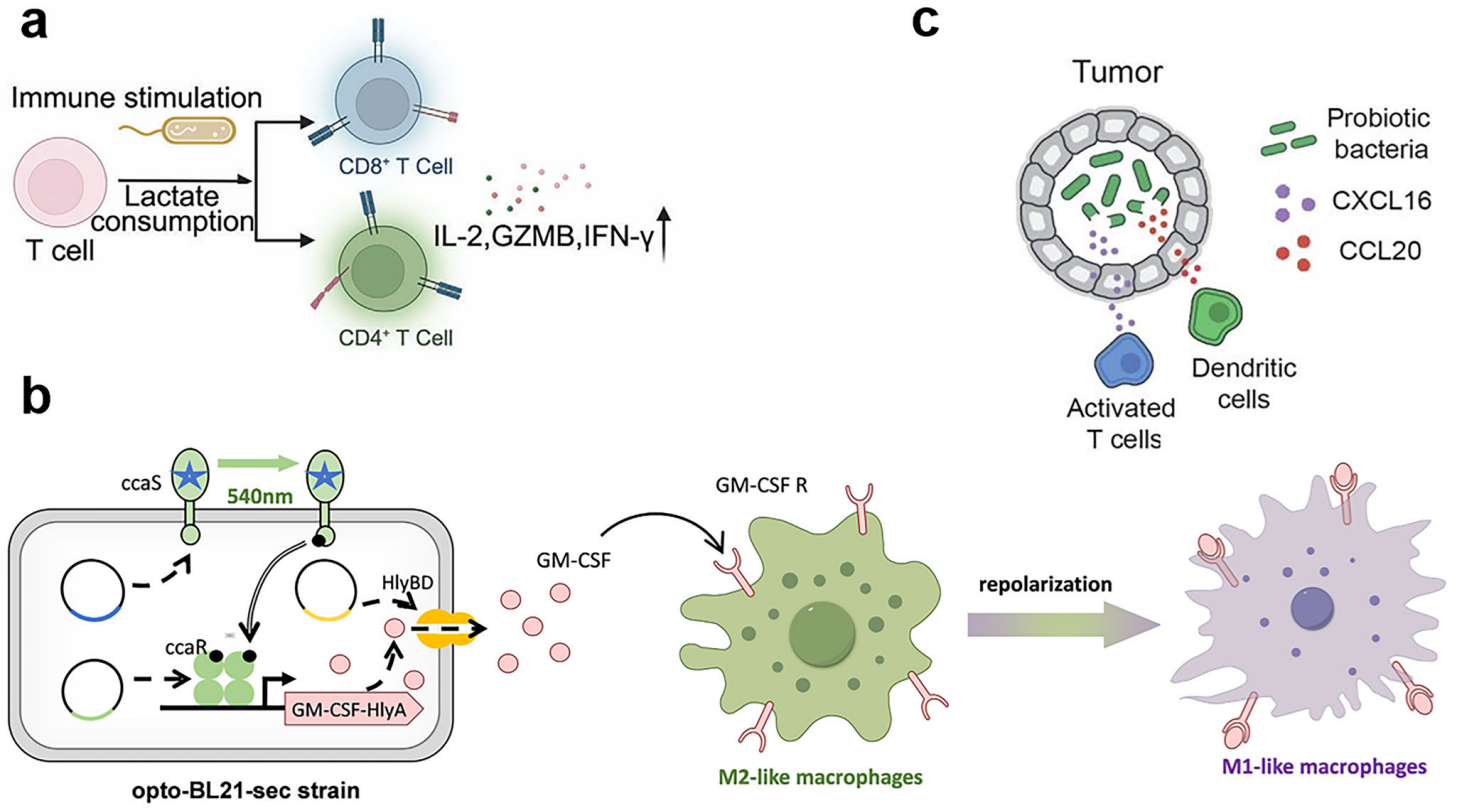
Bacteria‐mediated modulation or remodeling of the tumor microenvironment. a) Lactate consumption in tumor microenvironment by bacteria. *R. palustris* LAB‐1 was employed to metabolize intratumoral lactate into pyruvate via the native lactate dehydrogenase pathway, which reversed TME acidification and remodeled the tumor microenvironment. This metabolic reprogramming not only promoted the activation of CD4⁺ and CD8⁺ T cells but also upregulated immune effectors such as IL‐2, granzyme B, and IFN‐γ. Furthermore, it polarized macrophages from an M2 to M1 phenotype and enhanced T cell infiltration, ultimately leading to tumor growth suppression. The image is reproduced from ref.[29] The image is reproduced with permission from Ma *et al.*, *Advanced Science*, 2024, 36, adc2405930. 2024 Wiley‐VCH. [Reprinted under license number 6016991182106]. b) Reprogramming of local immune cell populations by engineered secretion of protein factor. *E. coli* BL21 (opto‐BL21‐sec) was engineered with a green‐light‐responsive ccaS/ccaR optogenetic system to secrete GM‐CSF upon 540 nm light stimulation. This induced sustained GM‐CSF secretion, reprogramming M2‐like TAMs into a pro‐inflammatory M1 phenotype, thereby suppressing tumor growth. Additionally, the engineered strain was designed to secrete hemolysin I, further enhancing its therapeutic potential. The image is reproduced from ref.[41] The image is reproduced with permission from Wang *et al.*, *Biomaterials*, 2025, 317, adc123037. 2025 Elsevier Ltd. [Reprinted under license number 6017001210068]. c) Recruitment of immune cells by engineered probiotic. *E. coli* Nissle 1917 (EcN) was engineered to express CXCL16 and CCL20, promoting the recruitment of dendritic cells and T cells to the tumor site, thereby modulating the immune landscape of the tumor microenvironment. Additionally, EcN was equipped with a SLC to release CXCL16 K42A, a CD8⁺ T cell recruiter, and CCL20, a pre‐dendritic cell recruiter, overcoming immune exclusion and enhancing immune infiltration. The image is reproduced from Ref ^[^ ^23^ ^]^.The image is reproduced from Thomas *et al.*, *Science Advances*, 2023, 9, adc9436. 2023 American Association for the Advancement of Science. [Reprinted under license number 6017001462644].

Bacteria not only possess the ability to selectively colonize tumor tissues but also can metabolize abnormally accumulated metabolites within the TME, thereby reversing its immunosuppressive characteristics and providing a novel strategy for bacteria‐mediated cancer therapy.^[^ ^62^, ^63^ ^]^ During metabolic reprogramming, solid tumors frequently undergo the Warburg effect, producing excessive lactate, which leads to a significantly elevated lactate concentration in the TME (10–30 mm) and a concomitant decrease in pH (6.5–6.9).^[^ ^64^, ^65^, ^66^ ^]^ This acidic and lactate‐rich environment has been shown to suppress antitumor immune responses through multiple mechanisms, including inhibition of cytotoxic T lymphocytes (CTL) and natural killer (NK) cell effector functions by acidification, disruption of metabolic homeostasis via inhibition of monocarboxylate transporter 1/4‐mediated lactate transport, and activation of thymus‐derived lymphocyte (T cell) apoptotic and exhaustion pathways such as caspase‐3/9 and programmed cell death protein 1 (PD‐1) upregulation.^[^ ^65^, ^67^, ^68^ ^]^ Certain facultative anaerobic bacteria, endowed with natural tumor‐targeting properties and high lactate‐metabolizing capacity, have been shown to convert lactate to pyruvate via lactate dehydrogenase or lactate oxidase, subsequently feeding into the tricarboxylic acid cycle for energy production, or alternatively converting lactate to acetyl‐CoA through the methylglyoxal pathway for biosynthesis.^[^ ^62^, ^69^ ^]^ For example, Ma et al. identified a high‐lactate‐metabolizing photosynthetic strain, *R. palustris* LAB‐1, which can selectively colonize hypoxic and lactate‐enriched tumor regions.^[^ ^29^ ^]^ Upon intratumoral administration in a 4T1 murine breast cancer model, *R. palustris* LAB‐1 efficiently metabolized tumor‐derived lactate into pyruvate, entering energy‐generating pathways and resulting in a marked reduction in intratumoral lactate levels and an elevation of local pH. These metabolic changes promoted the polarization of tumor‐associated macrophages from the immunosuppressive M2 macrophages (M2) to the pro‐inflammatory M1 macrophages (M1) phenotype, enhanced dendritic cell maturation, and increased cluster of differentiation 4 positive T cells (CD4^+^) and cluster of differentiation 8 positive T cells (CD8^+^)T cell infiltration and activation within the tumor (**Figure** **2**a). This study demonstrated the feasibility and effectiveness of leveraging naturally derived bacteria to reprogram tumor metabolism, remodel the TME, and enhance antitumor immunity for therapeutic benefit.

Besides hypoxia and lactate accumulation, the other frequently present alternation in the TME is the up‐regulation of immunosuppressive factors.^[^ ^48^, ^70^, ^71^ ^]^ This change can profoundly affect the phenotype and function of the infiltrated immune cells.^[^ ^70^ ^]^ Among them, macrophages are particularly sensitive to TME‐derived signals and can undergo polarization from a pro‐inflammatory, anti‐tumorigenic M1 phenotype to an immunosuppressive, tumor‐promoting M2 phenotype, resulting in the accumulation of tumor‐associated macrophages that contribute to immune evasion and tumor progression.^[^ ^72^, ^73^, ^74^ ^]^ By rational design, engineered living bacteria emerged as a promising tool to reprogram local immune cell populations, thereby remodeling the immunological landscape and enhancing anti‐tumor responses within the TME.^[^ ^19^ ^]^ For example, Wang et al. intratumorally administered engineered *E. coli* BL21‐sec equipped with a hemolysin I secretion system and a green‐light‐responsive optogenetic module (ccaS/ccaR system) into mice bearing 4T1 breast tumors. Under continuous illumination at 540 nm, granulocyte‐macrophage colony‐stimulating factor (GM‐CSF) was persistently secreted at high levels. GM‐CSF secretion promoted the repolarization of immunosuppressive M2‐like tumor‐associated macrophages toward a pro‐inflammatory M1 phenotype. As a result, the proportion of M1‐like tumor‐associated macrophages was significantly increased, which effectively inhibited tumor growth (Figure 2b).^[^ ^30^, ^41^ ^]^

Besides reprogramming the local immune cell populations, engineered living bacteria can recruit external immune cells to modulate the tumor microenvironment and further enhance anti‐tumor immunity.^[^ ^9^ ^]^ Tumors often establish a “cold” immune landscape due to the limited effector T cell infiltration through multiple mechanisms, including structural barriers, deficient chemokine signaling, metabolic abnormalities, and immunosuppressive networks.^[^ ^75^, ^76^ ^]^ Consequently, this cold tumor immune environment causes the reduced responsiveness to immunotherapies such as PD‐1/programmed death‐ligand 1 (PD‐L1) blockade.^[^ ^77^ ^]^ The recruitment and activation of immune cells, particularly CD8⁺ CTLs, has been widely recognized as a key approach to enhance antitumor immune responses.^[^ ^50^, ^78^ ^]^ In this context, engineered bacteria were programmed to locally deliver chemokines within tumors, enabling precise immune cell recruitment to overcome immune exclusion and reshape the TME toward an immunologically active state.^[^ ^9^, ^20^ ^]^ For example, Savage et al. engineered a synchronized lysis circuit (SLC) into probiotic *E. coli* Nissle 1917, which was further modified to express either an enhanced chemokine variant CXCL16^K42A or CCL20^[^ ^23^ ^]^. Upon intratumoral injection into multiple murine tumor models (A20, MC38, and EO771), the bacteria proliferated within the tumor and underwent density‐dependent lysis, releasing CXCL16^K42A and CCL20 to selectively recruit CD8⁺ T cells and precursor dendritic cells, respectively. This strategy enhanced antigen presentation and T cell priming, with significantly increased intratumoral infiltration of CD8⁺ T cells and dendritic cells, and therefore activated effector pathways including IFN‐γ and Granzyme B for tumor suppression (Figure 2c). Collectively, the study demonstrated that engineered bacteria can achieve controlled, localized, and efficient immune cell accumulation, offering a promising approach to overcome immune exclusion and reprogram the TME for effective cancer immunotherapy.

In summary, engineering bacteria to modulate the TME enables the localized, multifaceted intervention beyond conventional drug delivery. This strategy acts through metabolic modulation (e.g., lactate or arginine depletion^[^ ^29^, ^79^ ^]^), immune remodeling (e.g., Toll‐like receptor 5 (TLR5) activation via FlaB, SLIC‐driven IFN‐γ pulses^[^ ^80^, ^81^ ^]^), and physical modulation (e.g., vascular normalization, extracellular matrix degradation^[^ ^82^ ^]^). Incorporating signal‐responsive modules, such as hypoxia‐ or ultrasound‐triggered circuits, can further enhance precision, as shown with *S. typhimurium* DB1 and ROS‐generating *E. coli*.^[^ ^83^, ^84^ ^]^

### 2.3 Immunity System Activation

#### 2.3.1 Restoring Tumor Immunogenicity

Tumors are not merely the result of abnormal cellular proliferation and metabolic reprogramming that alter the immune landscape, but also a manifestation of impaired immune recognition and dysfunction.^[^ ^85^ ^]^ Tumor initiation and progression are often accompanied by immune evasion, compromised antigen presentation, exhaustion of effector T cells, and the abnormal enrichment of immunosuppressive cells such as regulatory T cells (Tregs) and M2‐polarized TAMs.^[^ ^76^, ^86^ ^]^ Therefore, reestablishing a potent antitumor immune response—particularly by activating and sustaining CD8⁺ cytotoxic T lymphocytes and innate immune cells—has become a critical therapeutic objective.

**Figure 3:**
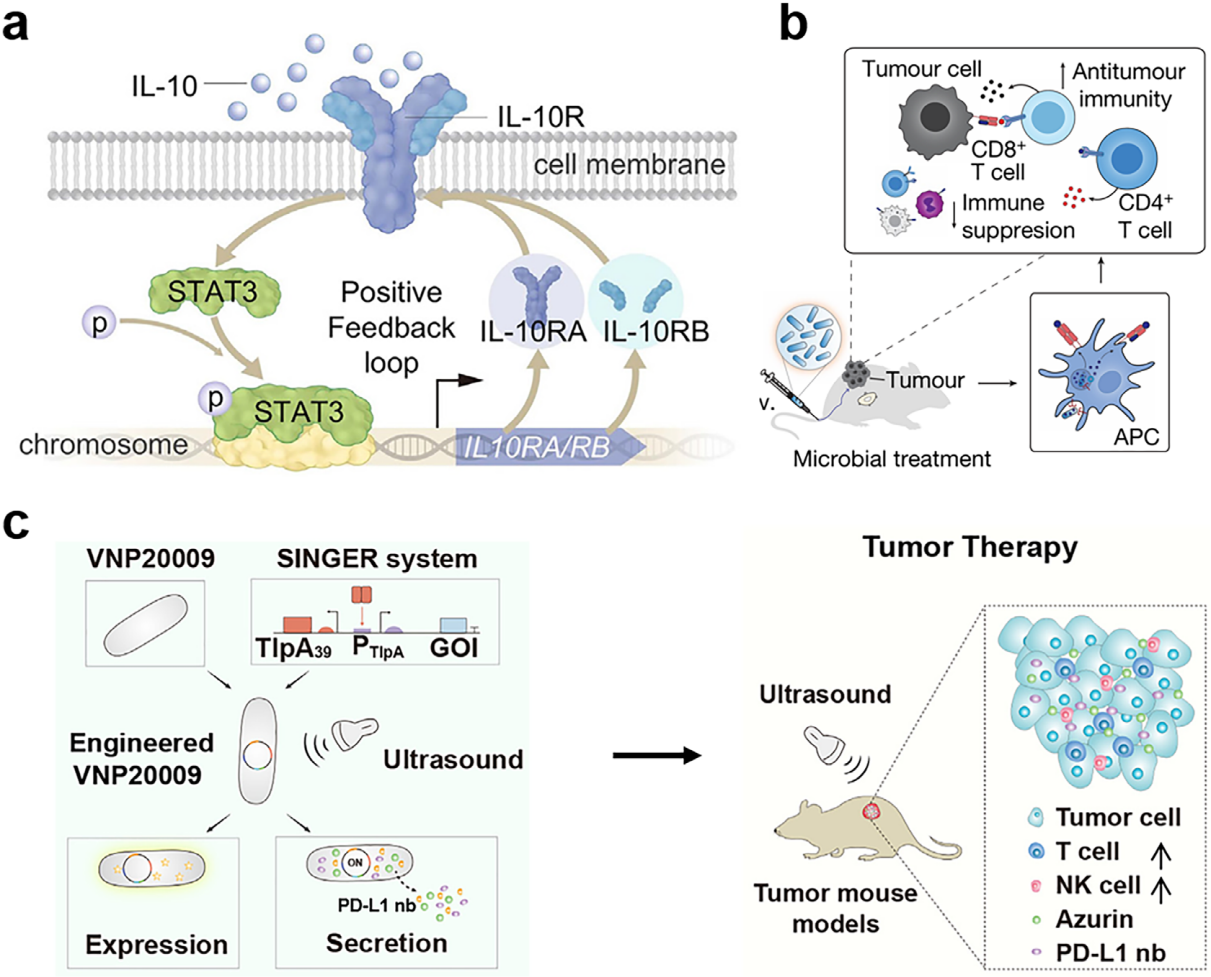
Activating the immune system by engineered bacteria to suppress tumor growth. a) Immunogenic response of bacterial colonization. *S. typhimurium* VNP20009 was engineered to selectively survive in hypoxic conditions. Following host phagocytosis, it activated the immune system and induced IL‐10 production. IL‐10 then triggered STAT3 signaling and upregulated IL‐10 receptor expression, forming a positive feedback loop. This response led to CD8⁺ T cell activation and tumor suppression. The image is reproduced from ref.[35] The image is reproduced with permission from Chang *et al.*, *Cell*, 2025, 188, adc1‐16. 2025 Elsevier Inc. [Reprinted under license number 6017000712717]. b) Delivery of specific tumor antigens by engineered probiotic. *E. coli* Nissle 1917 (EcN) was engineered to express tandem tumor‐specific neoantigen peptides derived from tumor‐associated mutated epitopes, promoting phagocyte uptake and activating CTLs to inhibit tumor growth. The engineered EcN continuously expresses these neoantigens under a constitutive promoter, enabling sustained antigen release that enhances cross‐presentation by dendritic cells, thereby activating CD8⁺/CD4⁺ T cells and stimulating Th1 immunity. The image is reproduced from ref.[32] The image is reproduced from Andrew *et al.*, *Nature*, 2024, 635, adc8038. 2024 The Author(s). Licensed under CC BY 4.0. https://doi.org/ 10.1038/s41586‐024‐08033‐4. c) Restoring tumor immunogenicity through local expression of PD‐L1 nanobody by engineered bacteria. *S. typhimurium* VNP20009 was engineered with a thermosensitive transcriptional system, which is activated by ultrasound‐induced mild hyperthermia to drive local expression of a PD‐L1 nanobody at tumor sites. This prevents immune evasion and activates antitumor immune responses, including T and NK cell activation. The image is reproduced from Ref ^[^ ^27^ ^]^.The image is reproduced with permission from Gao *et al.*, *Cell reports medicine*, 2024, 5, adc101513. 2024 Elsevier Inc. [Reprinted under license number 6017010735414].

Certain living bacteria offer unique advantages due to their sustained local immunostimulation capability.^[^ ^19^, ^20^ ^]^ For example, Zhang et al. developed a genetically engineered *S. typhimurium* strain, DB1, which exhibits potent anticancer effects through immune modulation. DB1 survives and proliferates only in hypoxic tumor environments, since an oxygen‐regulated *asd* gene caused the lysis of bacteria in oxygen‐rich tissues (**Figure** **3**a).^[^ ^35^ ^]^ The researchers discovered a hysteresis effect of interleukin‐10 receptor (IL‐10R), which described how DB1 modulated the tumor immunity. The introduction of DB1 increased the IL‐10R level and caused a high IL‐10R expression state (IL‐10R^hi^), and this state was maintained even after IL‐10 levels decreased. DB1 stimulates TAMs to secrete IL‐10, reinforcing the IL‐10R^hi^ state. Through IL‐10R^hi^, IL‐10 suppresses tumor‐associated neutrophil phagocytosis via signal transducer and activator of transcription 3 (STAT3) signaling, ensuring bacterial survival. Concurrently, IL‐10 activates exhausted CD8⁺ T cells (tumor‐resident memory‐like, CD62L^−^CD69⁺), boosting IFN‐γ production and cytotoxicity, which contributes to tumor elimination, recurrence prevention, and metastasis inhibition.

Bacteria are often recognized by the host immune system as foreign entities capable of inducing global immunogenic responses. Additionally, certain bacterial proteins can function as independent immune agonists that directly activate host immunity.^[^ ^87^, ^88^ ^]^ For example, FlaB, a flagellin protein produced during the growth of *Vibrio vulnificus*, is a natural agonist of TLR5.^[^ ^89^ ^]^ It has been shown to switch on the TLR5 ‐ myeloid differentiation primary response gene 88‐ (MyD88)‐nuclear factor kappa‐light‐chain‐enhancer of activated B cells (NF‐κB) signaling pathway in immune cells, leading to the elevated expression of pro‐inflammatory cytokines such as interleukin‐1β (IL‐1β) and tumor necrosis factor‐alpha (TNF‐α).^[^ ^90^ ^]^ In parallel, FlaB can also synergistically activate the NOD‐like receptor family pyrin domain containing 3 inflammasome by facilitating the caspase‐1–mediated cleavage of pro‐IL‐1β, thereby enhancing local inflammatory responses and innate immune activation.^[^ ^91^ ^]^ For instance, Nguyen et al. engineered an attenuated *S. typhimurium* strain (SAM‐FlaB) to express and secrete FlaB via the type III secretion system, and administered it into 4T1 murine breast tumor models. Following colonization within the tumor tissue, FlaB expression successfully triggered the repolarization of TAMs from an M2‐like immunosuppressive phenotype toward a pro‐inflammatory M1‐like state. The treatment simultaneously enhanced the activity of effector immune cells such as NK cells (Figure 1d).^[^ ^28^ ^]^ This dual modulation helped to reverse the immunosuppressive tumor microenvironment and restore antitumor immune responses.

#### 2.3.2 Activating Antigen‐Specific Immunity

Complementary to restoring the global immunity, the activation of tumor‐specific immunity offers a more targeted strategy for cancer treatment. This approach enables the precise recognition and selective elimination of tumor cells, while minimizing off‐target toxicity to healthy tissues.^[^ ^50^ ^]^ By programming bacteria to continuously express tumor‐specific antigens, a sustained and precise immune stimulation can be achieved within the host by activating tumor‐specific T cells.^[^ ^20^ ^]^ For instance, Redenti et al. developed a living bacterial vaccine platform based on a genetically engineered derivative of the probiotic *E. coli* Nissle 1917.^[^ ^32^ ^]^ This strain was designed to express tandem neoantigen peptides derived from tumor‐specific mutated epitopes, capable of simultaneously activating both CD8⁺ CTLs and CD4⁺ helper T cells (Figure 3b). Upon intravenous administration into mice bearing colorectal cancer (CT26) and melanoma (B16F10) tumors, the engineered *E. coli* Nissle 1917 colonized tumor tissues, synthesized, and released the neoantigens. Following their uptake by dendritic cells and macrophages, these antigens drove robust major histocompatibility complex (MHC) class I–mediated activation of CD8⁺ T cells, characterized by extensive proliferation and the secretion of IFN‐γ and Interleukin‐2 (IL‐2). Concurrently, MHC class II–mediated activation of CD4⁺ T cells provided essential helper signals to sustain the CD8⁺ T cell response, further amplifying a potent Th1‐type immune response marked by high levels of IFN‐γ and IL‐2 production. This combined strategy effectively suppressed tumor growth and established long‐term immunological memory, thereby conferring lasting protection against tumor recurrence.

As another example, Chen et al. engineered the skin commensal bacterium *Staphylococcus epidermidis* to express melanoma‐derived tumor antigens.^[^ ^24^ ^]^ Following topical application or stable colonization in B16 melanoma‐bearing mice, antigens produced by *S. epidermidis* were recognized and processed by cutaneous antigen‐presenting cells. This triggered activation of tumor‐specific CD8⁺ and CD4⁺ cytotoxic T cells, which migrated to tumor sites, exerted cytotoxic activity, and established durable immunological memory. This approach demonstrates engineered skin‐resident bacteria as a noninvasive platform for activating tumor‐specific immunity, offering a novel strategy for cancer.

#### 2.3.3 Blocking Immune Evasion

During the progression of solid tumors, cancer cells commonly evade immune surveillance by expressing immunoregulatory molecules such as PD‐L1, which interact with receptors on T cells to suppress their recognition of tumor‐specific antigens.^[^ ^50^ ^]^ This interaction leads to T cell dysfunction and exhaustion, ultimately resulting in immune evasion.^[^ ^86^ ^]^ IFN‐γ is a key immunomodulatory cytokine known to enhance antigen presentation, activate macrophages, and stimulate antitumor activity of both CD8⁺ T cells and NK cells.^[^ ^92^ ^]^ However, the systemic administration of IFN‐γ is frequently associated with significant toxicity, fluctuating serum concentrations, and poor tumor targeting.^[^ ^93^ ^]^ In contrast, the engineered microbes capable of tumor‐specific colonization and sustained IFN‐γ release may minimize the systemic toxicity while effectively boosting local immune responses to clear the tumor.

For example, Li et al. integrated a quorum‐sensing circuit into the genome of the probiotic strain *E. coli* Nissle 1917, in which the accumulation of the autoinducer 3OC6‐HSL at high cell densities activates the expression of phage‐derived E lysis protein, leading to bacterial cell lysis and the synchronized release of therapeutic payloads.^[^ ^30^ ^]^ Following intravenous administration into murine models of colorectal cancer (MC38 and CT26) and MHC‐I–deficient tumors, the modified *E. coli* strain selectively accumulated within tumor sites. Upon reaching a critical population density, the bacteria underwent synchronized lysis and release IFN‐γ protein (Figure 1b). The released IFN‐γ successfully up‐regulated MHC‐I expression on tumor cells, enhanced antigen presentation, and promoted the activation of both CD8⁺ cytotoxic T lymphocytes and CD4⁺ helper T cells. Concurrently, it stimulated NK cells to target MHC‐I–deficient tumor cells. This strategy demonstrated potent antitumor efficacy, highlighting the potential of engineered bacterial vectors to overcome tumor immune evasion mechanisms.

PD‐L1 antibody can specifically bind PD‐L1 molecules on tumor cells, blocking their interaction with PD‐1 receptors on T cells and disrupting this key immune checkpoint‐mediated evasion mechanism.^[^ ^50^ ^]^ This strategy reactivates tumor‐specific immunity and restores T cell effector functions. Building on this rationale, Gao et al. engineered the attenuated *S. typhimurium* strain VNP20009 for sonogenetically controlled expression of a PD‐L1 nanobody.^[^ ^27^ ^]^ The strain, delivered intravenously into multiple murine tumor models, incorporated the Synthetic Interruption of Genes by Excision and Replacement system: a temperature‐sensitive module based on the thermolabile repressor TlpA39. Low‐intensity pulsed ultrasound applied to the tumor site elevated local temperature to 39–40 °C. This caused a conformational change in TlpA39, de‐repressing the downstream promoter P_TlpA_ and inducing expression of the PD‐L1 nanobody fused to the Yersinia secretion tag YopE1–15 (Figure 3c). The secreted nanobody effectively blocked tumor immunosuppression in B16F10 melanoma, CT26 colorectal carcinoma, A20 lymphoma, and H22 hepatocellular carcinoma models. This reversed CD8⁺ T cell inhibition, restored cytotoxic activity, increased IFN‐γ production, and significantly suppressed tumor growth.

**Figure 4:**
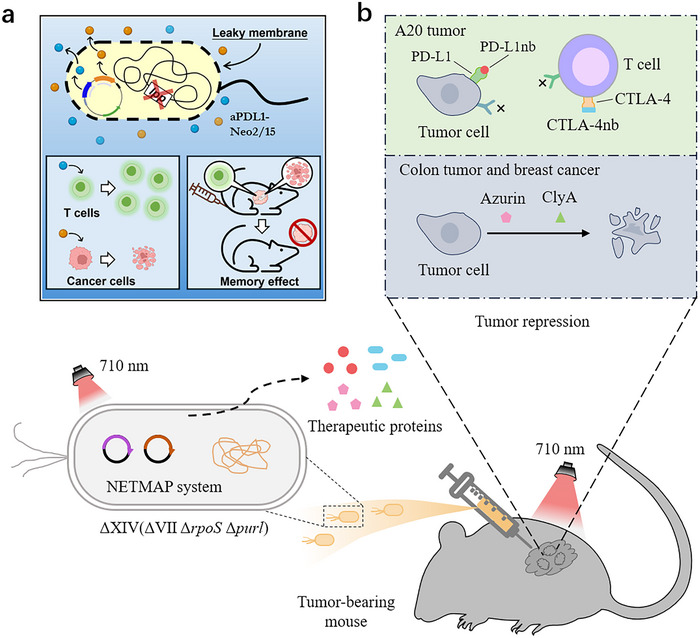
Engineering bacteria to express multiple effectors to block immune evasion and suppress tumors. a) Prevention of tumor immune evasion by drug fusion. The outer membrane–leaky *E. coli* strain Δlpp‐CIOP was engineered to produce a fusion of anti‐PD‐L1 nanobody and Neoleukin‐2/15. This engineered bacterium activates tumor CD8⁺ T cells, enhances tumor suppression, and induces durable immune memory. The image is reproduced from Ref ^[^ ^34^ ^]^.The image is reproduced with permission from Liu *et al.*, *Cell reports medicine*, 2024, 5, adc101793. 2024 Elsevier Inc. [Reprinted under license number 6017011071703]. b) Combined antibodies‐mediated immune activation. *S. enteritidis* 3934 ΔXIV, engineered with the NETMAP system, was designed to secrete PD‐L1 nanobodies, CTLA‐4 nanobodies, azurin, and ClyA under NIR light, thereby blocking immune evasion and activating CD8⁺ T cells and NK cells. The image is reproduced from ref.[37] The image is adapted from Qiao *et al.*, *Nature Cancer*, 2025, 6(4), adc612–628, redrawn based on conceptual content. This figure is an original reinterpretation and does not contain copyrighted material.

Similarly, Liu et al. engineered an attenuated *E. coli* Nissle 1917 strain (EcNΔlpp‐CIOP) by deleting the *lpp* gene to create a “leaky” outer membrane structure that facilitates protein release.^[^ ^34^ ^]^ Under arabinose‐inducible control, the strain expressed a chimeric fusion of an anti–PD‐L1 nanobody with Neoleukin‐2/15 (aPDL1‐Neo2/15) (**Figure** **4**a). Upon intravenous administration into murine tumor models, including colorectal cancer (MC38), mesothelin‐expressing colon adenocarcinoma (CT26‐MSLN), and melanoma (B16F10), the engineered bacteria effectively alleviated PD‐L1–mediated suppression of CD8⁺ cytotoxic T lymphocytes within the tumor microenvironment, thereby significantly inhibiting tumor growth.

Within tumor‐specific immune responses, PD‐L1 primarily suppresses cytotoxic T cell activity in the TME by binding PD‐1 receptors,^[^ ^94^ ^]^ while cytotoxic T‐lymphocyte associated protein 4 (CTLA‐4) inhibits early T cell activation through competitive B7 family of costimulatory molecules (B7) binding.^[^ ^95^ ^]^ These checkpoints synergistically establish profound immunosuppression.^[^ ^96^ ^]^ Dual blockade of PD‐L1/PD‐1 and CTLA‐4/B7 axes thus reactivates exhausted T cells and facilitates initial priming, enhancing antitumor efficacy.^[^ ^97^, ^98^ ^]^ Leveraging this strategy, Qiao et al. engineered attenuated *Salmonella enteritidis* (*S. enteritidis*) (3934 ΔXIV) for near‐infrared (NIR) light‐inducible protein expression. Following intratumoral administration in A20 lymphoma‐bearing mice, NIR irradiation elevated local temperature, triggering expression and outer membrane protein A ‐mediated secretion of PD‐L1/CTLA‐4 nanobodies^[^ ^37^ ^]^ (Figure 4b). These nanobodies simultaneously blocked both checkpoint pathways, enhancing T cell activation, restoring CD8⁺ T/NK cell cytotoxicity, and inducing proinflammatory cytokines (IFN‐γ, TNF‐α). Consequently, intratumoral Tregs and M2 macrophages decreased significantly, leading to the delayed primary tumor growth, distal tumor suppression as well as durable immune memory establishment.

Building on the role of GM‐CSF in promoting dendritic cell/macrophage accumulation and maturation to enhance antigen presentation and T cell priming,^[^ ^99^ ^]^ combined delivery with PD‐L1 and CTLA‐4 nanobodies targets immune initiation, activation, and effector checkpoints to reprogram the tumor microenvironment for sustained antitumor immunity. Accordingly, Gurbatri et al. engineered an orally administered *E. coli* Nissle 1917 probiotic that colonized tumor surfaces in a murine colorectal adenoma model.^[^ ^25^ ^]^ Upon reaching quorum density, the strain underwent synchronized lysis, locally releasing GM‐CSF, PD‐L1 nanobody, and CTLA‐4 nanobody. This synergistic triad activated local/systemic antitumor T cell responses, significantly reduced tumor burden, and increased tumor immunogenicity. The study establishes engineered probiotics as “living immune‐modulating factories” for precise intratumoral delivery of combinatorial therapeutics, offering a safe, modular, and orally deliverable microbiome‐based immunotherapy platform.

In conclusion, engineered bacteria offer innovative strategies to restore tumor immunogenicity and overcome immune evasion.^[^ ^21^, ^100^ ^]^ Compared to conventional approaches, rational engineering of bacteria can endow the system with unique advantages, including tumor‐specific colonization, sustained local delivery, and genetic programmability for multi‐stage immune modulation.^[^ ^22^ ^]^

## 3 Future Outlook

Synthetic biology is revolutionizing cancer therapy by enabling the development of engineered bacteria as sophisticated living therapeutics. These systems offer significant advantages over conventional treatments, including enhanced precision and reduced side effects.^[^ ^9^, ^19^ ^]^ The superior targeting capacity of engineered microbes is partially attributed to their chemotactic behavior and environmental adaptability.^[^ ^19^ ^]^ For example, the photosynthetic bacterium *R. palustris* LAB‐1 selectively metabolizes tumor‐derived lactate to remodel the immunosuppressive tumor microenvironment,^[^ ^29^ ^]^ while *S. typhimurium* strain SAM‐FlaB activates anti‐tumor M1 macrophages via the TLR5/NF‐κB signaling axis.^[^ ^28^ ^]^ These natural targeting capabilities can be further augmented through rational engineering; genetic circuits, such as those suppressing bacterial growth under aerobic conditions, ensure proliferation is restricted to tumors and spares healthy organs.^[^ ^35^ ^]^ This precise localization enables effective local immune modulation. Furthermore, engineered bacteria achieve sustained therapeutic persistence through intrinsic self‐replication and programmable control mechanisms. A prime example is *E. coli* Nissle 1917, engineered with a quorum‐sensing circuit for the spatiotemporal release of IFN‐γ.^[^ ^30^ ^]^ Collectively, these features provide engineered bacteria with unique and powerful advantages for maintaining high local drug concentrations and enabling long‐term immune activation against cancer.

Despite its promise, the clinical translation of engineered bacteria faces significant challenges. A primary concern is balancing immunogenicity and safety. For example, bacterial lipopolysaccharide activates host TLR4, triggering cytokine production, including immunosuppressive IL‐10. While protective against excessive inflammation physiologically,^[^ ^101^ ^]^ elevated tumor IL‐10 inhibits dendritic cell maturation (via downregulation of MHC‐II, CD80, CD86), impairing T cell activation and compromising antitumor efficacy.^[^ ^9^, ^20^, ^102^ ^]^ Precise dose control is also complicated by the bacteria's living, proliferative nature.^[^ ^9^ ^]^ Furthermore, host microbial ecology and immunological memory can cause inconsistent outcomes through competition with commensals.^[^ ^103^ ^]^ Long‐term safety concerns include risks of systemic infection, chronic inflammation, and excessive immune activation.^[^ ^9^, ^104^ ^]^

To mitigate these challenges, several safety engineering strategies have been implemented. Attenuation of virulence remains a foundational approach, whereby deletion of key pathogenic genes significantly reduces toxicity while preserving antitumor functions; for example, knocking out the ppGpp synthesis genes (*relA* and *spoT*) in *S. typhimurium* increases its LD_50_ by 10^5^–10^6^ fold compared to the wild‐type strain while maintaining the ability to stimulate proinflammatory cytokines such as IL‐1β, interleukin‐18, and TNF‐α within the tumor microenvironment.^[^ ^105^ ^]^ Similarly, the well‐studied strain *S. typhimurium* VNP20009, which harbors deletions in *msbB* and *purI* genes, demonstrates markedly reduced endotoxicity and has been evaluated in human clinical trials.^[^ ^106^, ^107^ ^]^ Bio‐containment strategies, such as the development of inducible suicide circuits, may ensure bacterial self‐elimination post‐delivery.^[^ ^11^, ^108^ ^]^ Furthermore, immune modulation strategies like transient antigen cloaking using engineered polysaccharide capsules help reduce undesired immune responses while preserving therapeutic efficacy, offering a balanced approach between immunogenicity and tolerability.^[^ ^22^, ^109^ ^]^

Translating engineered bacterial cancer therapies to the clinic also faces practical and regulatory hurdles. A key challenge is the immature regulatory landscape for living microbial therapeutics, requiring early agency engagement and case‐by‐case toxicology assessments due to a lack of standardized guidelines.^[^ ^110^ ^]^ In parallel, producing these therapies under Good Manufacturing Practice (GMP) conditions poses substantial technical hurdles. Because live bacteria cannot undergo terminal sterilization by heat or filtration, manufacturing must be conducted in dedicated aseptic facilities with rigorous contamination control.^[^ ^9^ ^]^ Moreover, scalability remains a significant concern. Transitioning from laboratory‐scale cultures to clinical‐grade production requires maintaining genetic stability and consistent bioactivity across batches, and the manufacturing process often must be customized to match specific dosing regimens and delivery routes.^[^ ^110^, ^111^ ^]^ Therefore, the successful clinical translation of engineered bacterial therapies critically depends on addressing these interconnected challenges in regulation, GMP‐compliant production, safety assurance, and scalable biomanufacturing.

Despite persistent challenges, several early‐phase clinical studies have provided preliminary evidence supporting the feasibility of applying engineered bacteria in cancer therapy. A representative example is SYNB1891, an engineered probiotic *E. coli* Nissle 1917 strain designed to express a stimulator of interferon genes (STING) agonist under hypoxic conditions. In a recent Phase I clinical trial (NCT04167137), intratumoral administration of SYNB1891—either alone or in combination with the immune checkpoint inhibitor atezolizumab—was shown to be safe and well tolerated, while effectively activating STING signaling and inducing T cell responses in patients with advanced malignancies.^[^ ^112^ ^]^ Another milestone study involved the intravenous delivery of the attenuated *S. typhimurium* strain VNP20009 in patients with metastatic melanoma, which confirmed successful tumor colonization and the induction of systemic cytokine responses.^[^ ^106^ ^]^ These two cases provide critical clinical insights into the safety, tumor‐targeting capacity, and immunomodulatory potential of living bacterial therapeutics in humans, representing an important step toward the clinical translation of programmable bacterial cancer therapies.

In the future, the successful transition of bacterial therapeutics from bench to bedside will depend on a deeper understanding of the working mechanism, the establishment of comprehensive regulatory frameworks, and the integration of diverse technological platforms. Understanding the mechanistic basis of bacterial cancer therapy is crucial for rational optimization of safety and efficacy. The construction of standardized, modular bacterial strain libraries—comprising functional units for immune activation, metabolic intervention, and drug delivery—would be a solid step toward personalized medicine.^[^ ^113^ ^]^ The incorporation of intelligent and visualized control systems has enabled real‐time monitoring and remote modulation of therapeutic processes. The development of GMP‐compliant manufacturing protocols and robust environmental risk assessment systems, including inducible suicide mechanisms, is essential to ensure the safety, controllability, and sustainability of clinical applications.

## Conflict of Interest

The authors declare no conflict of interest.

## Author Contributions

J.C. wrote, reviewed, and edited the original draft. J.C., Y.C., W.Y., and J.Z. reviewed and edited the manuscript. G.W. reviewed and edited the manuscript. Z.D. conceived the work, wrote and reviewed the draft.

## Data Availability

Data sharing is not applicable to this article as no new data were created or analyzed in this study.
